# Differential Effects of Chronic Ingestion of Refined Sugars versus Natural Sweeteners on Insulin Resistance and Hepatic Steatosis in a Rat Model of Diet-Induced Obesity

**DOI:** 10.3390/nu12082292

**Published:** 2020-07-30

**Authors:** Marion Valle, Philippe St-Pierre, Geneviève Pilon, André Marette

**Affiliations:** 1 Quebec Heart and Lung Institute Research Centre, Faculty of Medicine, Laval University, Quebec City, QC G1V 4G5, Canada; marion.valle@outlook.fr (M.V.); philippe.st-pierre@sn.ulaval.ca (P.S.-P.); genevieve.pilon@criucpq.ulaval.ca (G.P.); 2 Institute of Nutrition and Functional Foods, Laval University, Quebec City, QC G1V 0A6, Canada

**Keywords:** sucrose, fructose, syrup, glucose metabolism, non-alcoholic fatty liver disease (NAFLD), metabolic syndrome

## Abstract

While the detrimental effect of refined sugars on health has been the subject of many investigations, little is known about the long-term impact of natural sweeteners on metabolic disorders. In this study we compared the metabolic responses to chronic ingestion of refined sugars compared to various natural sweeteners in diet-induced obese rats. Wistar rats were fed a high-fat high-sucrose diet (HFHS) for 8 weeks and daily gavaged with a solution containing 1 g of total carbohydrates from refined sugar (sucrose or fructose) or six different natural sugar sources, followed by assessment of glucose homeostasis, hepatic lipid accumulation, and inflammation. While glucose tolerance was similar following treatments with refined and natural sugars, lowered glucose-induced hyperinsulinemia was observed with fructose. Consumption of fructose and all-natural sweeteners but not corn syrup were associated with lower insulin resistance as revealed by reduced fasting insulin and homeostatic model assessment of insulin resistance (HOMA-IR) compared to sucrose treatment of HFHS-fed rats. All-natural sweeteners and fructose induced similar liver lipid accumulation as sucrose. Nevertheless, maple syrup, molasses, agave syrup, and corn syrup as well as fructose further reduced hepatic IL-1β levels compared to sucrose treatment. We conclude that natural sweeteners and especially maple syrup, molasses, and agave syrup attenuate the development of insulin resistance and hepatic inflammation compared to sucrose in diet-induced obese rats, suggesting that consumption of those natural sweeteners is a less harmful alternative to sucrose in the context of obesity.

## 1. Introduction

Obesity is a worldwide epidemic associated with many metabolic disorders such as insulin resistance, increasing the risk of developing metabolic syndrome (MetS), non-alcoholic fatty liver disease (NAFLD), type 2 diabetes (T2D), and cardiovascular disease (CVD) [1,2]. Obesity and associated metabolic disorders have arisen in parallel with the increased adoption of the Western lifestyle which is in part characterized by a diet rich in fat, refined carbohydrates, and generally low in fibers [3]. Among these dietary factors, added sugars—defined as sugars and syrups added to foods and beverages during preparation or processing—have been particularly pointed out. Excessive consumption of added sugars (>10% of total energy intake) has been demonstrated to play a key role in the obesity pandemic [4,5]. Consequently, several public health organizations recommend limiting its intake [6]. Nevertheless, cutting down on sugar consumption is a daunting task as it is easy to underestimate the amount of added sugars consumed daily. Indeed, two-thirds of packaged foods contain added sugars (e.g., sucrose, glucose, or fructose) to enhance flavor or to extend shelf life [7]. Sweets and soft drinks typically contain high levels of added sugar but even “so-called” healthier food products, such as granola bars, cereals, and yoghurt, can also contain high amounts of added sugars.

High intake of fructose is currently pointed out as the principal culprit for the rapid rise of metabolic diseases associated with obesity. Indeed, excessive consumption of fructose has been reported to induce hepatic lipid accumulation [8], dyslipidemia [9,10], and insulin resistance [9,10]. Fructose has also been shown to be more detrimental than glucose in promoting visceral fat accumulation [11,12], liver steatosis [13], dyslipidemia [11], and insulin resistance [11,12,13]. These data are concerning, knowing that fructose represents about 50% of all added sugar consumed [7]. Furthermore, few studies suggest that free fructose may have more severe adverse metabolic effects than bounded fructose when comparing the effect of an equivalent mix of fructose and glucose to sucrose [14,15]. However, it is important to point out that most studies showing detrimental effects of fructose have used relatively high doses. In studies using moderate doses more compatible with human consumption, fructose did not alter metabolic health to a greater degree than equivalent doses of glucose or sucrose as reviewed in two recent meta-analyses [16,17], suggesting that fructose might be more detrimental than other sugars only when over consumed.

Previous studies have shown that consumption of natural sweeteners compared to refined sugar can improve metabolic health. It was previously reported that honey consumption limits weight gain, improves dyslipidemia, and reduces glycemia in both human [18,19] and rats [20,21]. Moreover, both maple syrup [22,23] and agave syrup [24] intake has been shown to improve glucose homeostasis compared to sucrose. Yet, honey is mostly composed of an equal mix of fructose and glucose, maple syrup is 97% sucrose and agave 90% fructose. However, in addition to their carbohydrate content, natural sweeteners are also enriched with many other molecules that may confer potential health benefits, such as phytohormones, minerals, and polyphenols [25,26,27,28], that may counteract the detrimental metabolic effects of the high sugars they contain. Indeed, honey contains about 181 substances (such as polyphenols) that have been reported for their beneficial impacts on hyperglycemia and hypertension in addition to antimicrobial properties [25]. As for maple syrup, it contains many interesting polyphenols, such as lignans and a unique phenolic molecule named quebecol [26], as well as phytonutrients and minerals [27,28]. Agave syrup also contains polyphenols and phytonutrients, but to a lesser extent [28]. Molasses has been shown to contain even higher concentrations of polyphenols than the aforementioned natural sweeteners [28] and has already been considered as a potential supplement for iron deficiency [29].

In a previous study, we compared several metabolic parameters following a single ingestion of an equivalent dose of carbohydrate from either glucose or specific natural sweeteners [28]. We showed the potential benefits of maple syrup, molasses, and agave syrup on glycemic and insulinemic responses, as well as on the secretion of hormones associated with food ingestion such as amylin and gastric inhibitory polypeptide. However, the potential metabolic benefits of chronic intake of low concentrations of natural sweeteners in the context of diet-induced obesity have not yet been investigated. Therefore, the objective of the current study was to compare in a rat model of diet-induced obesity the metabolic impact of low intake (4% of total energy) of chronic isocaloric carbohydrate consumption from sucrose to those from natural sweeteners as well as from fructose, as the data regarding its long-term effect on metabolic disorders remains conflicting considering the high doses that have been used in previous studies.

## 2. Materials and Methods

### 2.1. Sweeteners

The sweeteners used in the current study were previously characterized for their carbohydrate and phytochemical contents [28]. Maple syrup used in this study was a gift from the Québec Maple Producers. All other sweeteners, molasses (Grandma; Saint-John, NB, Canada), organic brown rice syrup (Sweet Dreams; Richvale, CA, USA), organic blue agave syrup (Wholesome Sweeteners; Sugar Land, TX, USA), golden corn syrup (Crown; Memphis, TN, USA), and pure natural honey (McCormick; London, ON, Canada) were purchased at a local grocery. Fructose was purchased from Sigma-Aldrich (Saint-Louis, MO, USA).

### 2.2. Experimental Design 

Wistar male rats (*n* = 108, Charles River Inc., Wilmington, MA, USA) weighing 250–300 g were housed two animals per cage in a temperature- and humidity-controlled room (21 ± 2 °C, 35–40%), with a daily 12:12 h light–dark cycle (lights on at 07:00 am). After 1 week of acclimation on a standard chow diet (Rodent Chow #2018 Harlan Teklad, Harlan Laboratories, Indianapolis, IN, USA), animals were randomly assigned to dietary groups, with ad libitum food and deionized water. Rats were fed a high-fat high-sucrose diet (HFHS; Teklad TD.130964 adj. kcal diet (66% kcal from fat; 19.8% lard, 19.8% corn oil; and 19% kcal from carbohydrates with sucrose as the only source of carbohydrate calories)) for 8 weeks. Animals received a daily gavage with an equivalent quantity of 1 g of total sugar (4% of total energy) from either maple syrup, molasses, brown rice syrup, agave syrup, corn syrup, honey, fructose, or a control solution of sucrose. The corresponding intake of 1 g of added sugar in total energy was calculated using the following equation: % of total energy as added sugar = (calories from 1g of carbohydrate (kcal) × 100)/daily total energy intake (kcal). One group of rats received a standard chow diet and daily gavage with sucrose, only as a reference group to determine the impact of the HFHS diet on obesity and metabolic parameters. Body weight gain and energy intake were assessed weekly. Food efficiency was calculated using the following equation: Food efficiency = (Body weight (g)/Food intake (g)) × 100. After 8 weeks of HFHS feeding, animals were fasted for 6 h and anaesthetized in chambers saturated with isoflurane and then sacrificed by cardiac puncture. Blood was drawn in ethylenediamine tetraacetic acid (EDTA)-treated tubes and immediately centrifuged to collect plasma. Liver, pancreas, inguinal, epididymal, and brown fat tissues were carefully collected, weighed, and snap-frozen in liquid nitrogen. The animal protocol met the guidelines of the Canadian Council on Animal Care and was approved by the Animal Care Committee of Laval University (CPAUL-111).

### 2.3. Indirect Calorimetry

After 6 weeks of diet treatments, 8–10 rats from each group were randomly placed in individual metabolic cages (Columbus Instruments, Columbus, OH, USA) to evaluate their energy expenditure and respiratory exchange ratio by measuring oxygen consumption and carbon dioxide production. Rats had free access to water and their respective diet during 24 h of acclimation prior to 48 h measurements. The temperature of the calorimetric room was set to 28 °C to keep the rats at thermoneutrality. Values were expressed per metabolic body weight, according to Kleiber’s law (body weight^0.75^) [30].

### 2.4. Oral Glucose Tolerance Test

Three days prior to sacrifice, after a 12 h fast, rats received an oral gavage of 2 g/kg of body weight solution of 50% dextrose. Blood samples were taken from the saphenous vein using EDTA-coated capillary tubes before (0 min), as well as 15, 30, 60, 90, and 120 min after the glucose administration. Glycemia was determined at each time point using a calibrated hand-held glucometer. The plasma was separated by centrifugation and stored at −80 °C until further analysis.

### 2.5. Biochemistry

Insulin levels were measured using ultrasensitive rat insulin (80-INSRTU-E 10) enzyme-linked immunosorbent assay (ELISA) kits (Alpco, Salem, NH, USA). The areas under the curve (AUC) for glucose and insulin were calculated using GraphPad Prism 5 for Mac OS X (GraphPad Software, San Diego, CA, USA), using the value at *t* = 0 min of each animal as baseline. The homeostasis model assessment of insulin resistance (HOMA-IR) index was calculated based on the following formula: fasting insulinemia (μUI/mL) × fasting glycemia (mM)/22.5. Liver triglyceride (TG) and total cholesterol contents were measured after chloroform–methanol extraction [31] with colorimetric kits (Randox Laboratories, Crumlin, UK). Cytokines interleukin-1 beta (IL-1β), interleukin-6 (IL-6), tumor necrosis factor alpha (TNF-α), and the chemiokine regulated on activation normal T-cell expressed (RANTES) were assessed in liver lysates (900 μg/mL protein in phosphate-buffered saline (PBS) containing 1% Nonidet P-40 (NP-40) and 0.01% protease inhibitor cocktail) by using a Bio-plex multiplex and Bio-plex 200 system (Biorad Laboratories, Hercules, CA, USA).

### 2.6. Liver Histology

Livers sections were fixed in 4% paraformaldehyde and then embedded in paraffin. Paraffin sections of 4 μm were stained with Hematoxylin and Eosin (H&E). Images were obtained by using the AxioObserverZ1 microscope (Zeiss, Jena, Germany) equipped with a 20× objective (numerical aperture 0.8) for qualitative assessment of lipid accumulation.

### 2.7. Statistical Analyses 

The biological effects of the HFHS-sucrose versus chow-sucrose (reference for HFHS-induced obesity) were assessed using Student’s *t*-tests. The effects of the natural sweeteners and the fructose compared to the sucrose control were assessed using one-way ANOVA with Dunnett post-hoc test. When equivalent variance test failed, non-parametric tests were used (Mann–Whitney test for *t*-test and Kruskal–Wallis test for ANOVA followed by Dunn’s post-hoc test). Comparisons over the course of the experiment were made using two-way repeated-measures ANOVA with Bonferoni post-hoc test. Significant differences (*p* < 0.05) between groups were reported. Statistical analyses were performed using GraphPad Prism 8 (GraphPad Software, San Diego, CA, USA). Results are presented as mean ± SEM.

## 3. Results

### 3.1. Effects of Added Refined and Natural Sweeteners on Energy Intake, Body Weight Gain, and Adiposity

**Figure 1:**
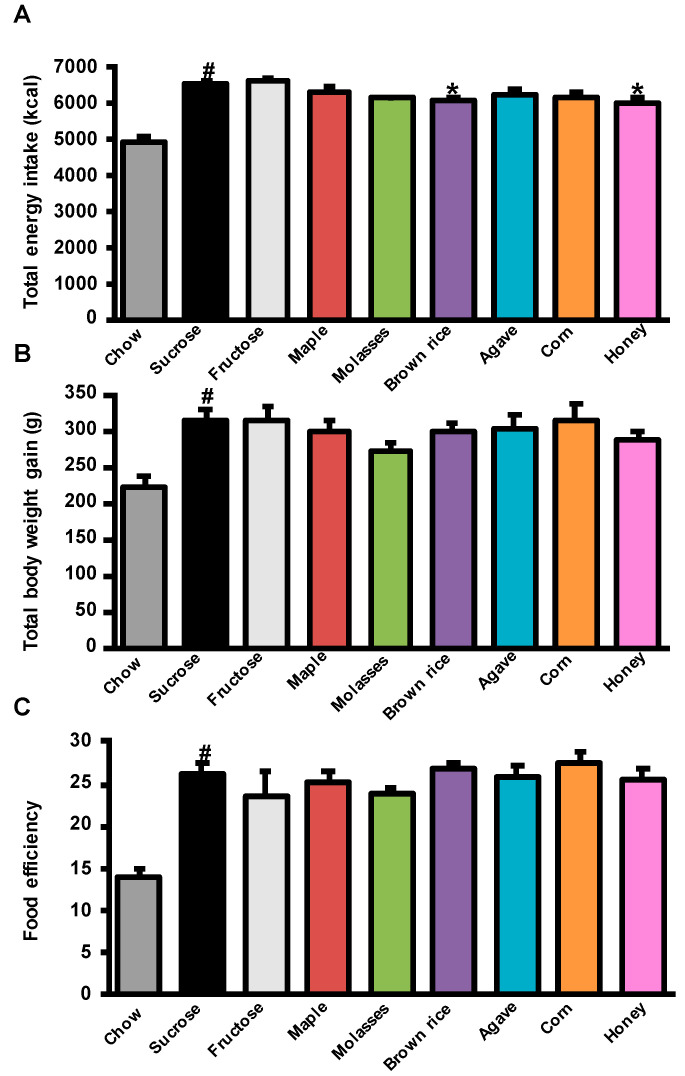
Refined sugars and natural sweeteners lead to similar obesity. Energy intake, body weight gain, and food efficiency in Wistar male rats treated with refined sugars and natural sweeteners for 8 weeks. (**A**) Total energy intake; (**B**) total body weight gain; (**C**) food efficiency. Data represent mean ± SEM, *n* = 10–12 rats. # *p* < 0.05, *t*-test for chow-sucrose (reference for high fat high sucrose (HFHS)-induced obesity) versus HFHS-sucrose. * *p* < 0.05, one-way ANOVA for fructose and natural sweeteners versus HFHS-sucrose.

The impact of the different refined sugars and natural sweeteners on energy intake, body weight gain, and adiposity are reported in Figure 1 and Table 1. HFHS-sucrose-treated rats consumed more energy and had higher food efficiency than the chow-sucrose-treated rats leading to greater body weight gain (Figure 1A–C). This was associated with increased visceral, subcutaneous, and brown adipose tissue weights (Table 1). Among the HFHS-fed groups, we observed a slight decrease in energy intake upon treatment with brown rice syrup and honey compared to the sucrose-treated group (Figure 1A). Nevertheless, this effect did not translate into significant changes in body weight gain (Figure 1B) and the food efficiency ratio was similar between the groups (Figure 1C).

Furthermore, there was no difference in visceral and subcutaneous adipose tissue weights between treatment groups but brown adipose tissue (BAT) weight was significantly lower in the maple syrup-, molasses-, and honey-treated rats than the sucrose-treated rats (Table 1). Fructose treatment affected neither energy intake nor body weight gain, final body, and organ weights (Figure 1A,B and Table 1). In addition, neither natural sweeteners nor fructose treatments impacted energy expenditure or the respiratory exchange ratio as measured by indirect calorimetry (Table 2).

### 3.2. Effects of Added Refined and Natural Sweeteners on Diet-Induced Insulin Resistance

**Figure 2:**
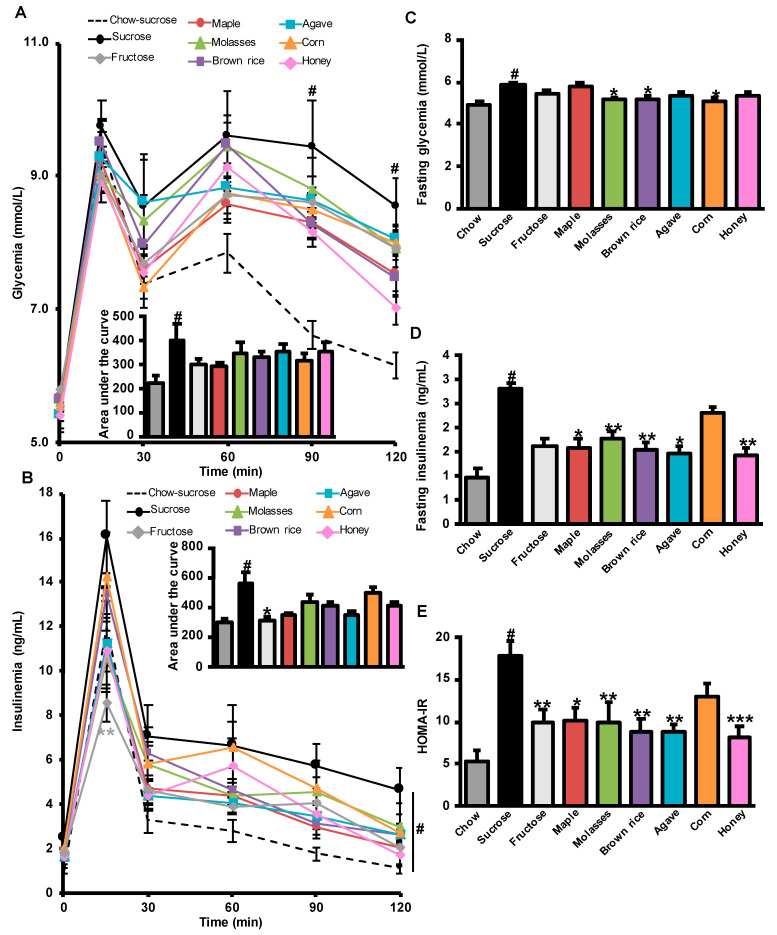
Fructose and natural sweeteners limit insulin resistance. Glucose and insulin homeostasis in male Wistar rats treated with refined sugars and natural sweeteners for 8 weeks. (**A**) Glycemic excursion curves during oral glucose tolerance test (OGTT) with their corresponding area under the curve of glucose. (**B**) Insulinemic excursion curves during OGTT and their corresponding area under the curve. (**C**) Fasting glycemia at sacrifice. (**D**) Fasting insulinemia at sacrifice. (**E**) Homeostatic model assessment of insulin resistance (HOMA-IR) at sacrifice. Data represent mean ± SEM, *n* = 10–12 rats. # *p* < 0.05, *t*-test for chow-sucrose (reference for HFHS-induced obesity) versus HFHS-sucrose. * *p* < 0.05, ** *p* < 0.01, *** *p* < 0.001, one-way ANOVA for HFHS fructose and natural sweeteners versus HFHS-sucrose. ** *p* < 0.01, two-way repeated-measures ANOVA for comparisons over the course of the OGTT in (**A**,**B**).

To compare the long-term effect of natural sweeteners and fructose versus that of sucrose on glucose homeostasis, rats were subjected to an oral glucose tolerance test (OGTT) after 8 weeks of treatment. As expected, the HFHS-fed control sucrose group showed clear glucose intolerance and hyperinsulinemia compared to the control sucrose group fed a chow diet (Figure 2A,B). However, no statistical differences were observed in the global glycemic response among all the HFHS groups, as depicted by the AUC (Figure 2A). In response to the glucose challenge, fructose-treated HFHS rats displayed a lower insulin peak at 15 min and showed improved overall insulin response (AUC) compared to the sucrose HFHS control group (Figure 2B). HFHS-sucrose-treated animals had both higher fasting glycemia and insulinemia than chow-sucrose-treated group and consequently higher insulin resistance as shown by higher HOMA-IR. Rats treated daily with molasses, brown rice, and corn syrups were found to display an improved fasting glycemia (Figure 2C). Moreover, when compared to sucrose, fasting insulinemia, and insulin resistance were improved by all-natural sweeteners, with the notable exception of corn syrup (Figure 2D,E). Interestingly, daily treatment with fructose was also found to tend to attenuate fasting insulin (*p* = 0.058) and to reduce insulin resistance as compared to an equivalent amount of sucrose (Figure 2D,E).

### 3.3. Effects of Added Refined and Natural Sweeteners on Liver Steatosis and Inflammation

**Figure 3:**
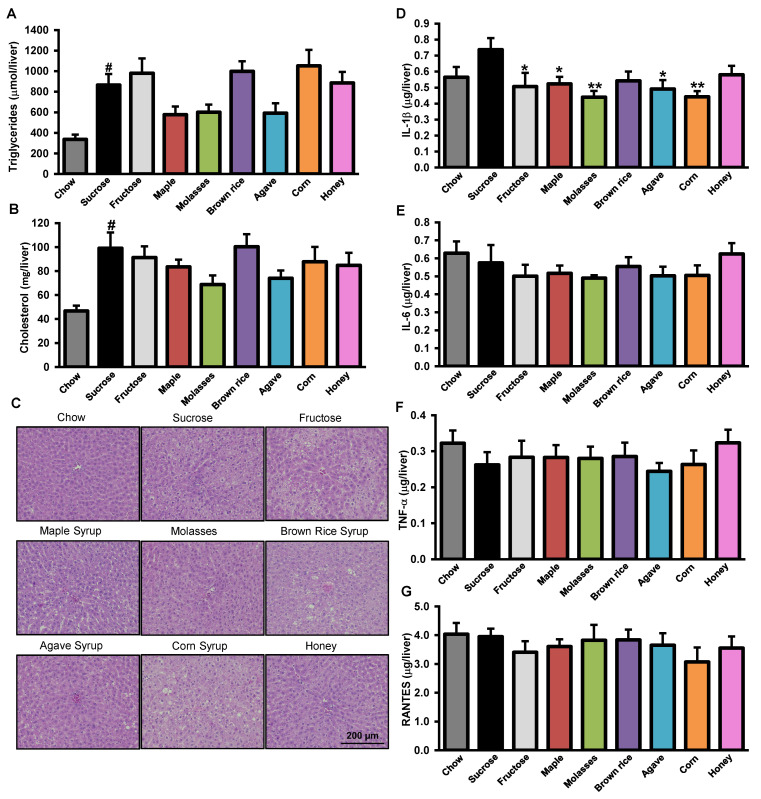
Natural sweeteners alleviated liver steatosis and inflammation. Liver steatosis, cholesterol content, and inflammation in male Wistar rats treated with refined sugars and natural sweeteners for 8 weeks. (**A**) Liver triglyceride TG content. (**B**) Liver cholesterol content. (**C**) Representative pictures of liver lipid droplets stained with H&E. (**D**) IL-1β in liver. (**E**) IL-6 in liver. (**F**) TNF-α in liver. (**G**) RANTES in liver. Data represent mean ± SEM. (**A**) and (**B**), *n* = 10–12 rats. (**C**), *n* = 6 rats. (**D**–**G**), *n* = 7–9 rats. # *p* < 0.05, *t*-test for chow-sucrose (reference for HFHS-induced obesity) versus HFHS-sucrose. * *p* < 0.05, ** *p* < 0.01, one-way ANOVA for HFHS-fructose and -natural sweeteners versus HFHS-sucrose.

As NAFLD is linked with obesity and insulin resistance [32], we therefore investigated the impact of the natural sweeteners as well as pure fructose on liver homeostasis. We first determined hepatic lipid accumulation using a biochemical method supported by qualitative histological images (Figure 3A–C). As expected, chronic sucrose gavage to HFHS-fed rats increased total hepatic TG (Figure 3A) and cholesterol content (Figure 3B), also evidenced by increased lipid droplet accumulation on H&E-stained liver sections (Figure 3C), compared to sucrose-treated chow-fed rats. Among HFHS-fed groups, the differences observed between both the TG and the cholesterol levels did not reach statistical significance (Figure 3A,B). Interestingly, rats daily gavaged with pure fructose did not accumulate more hepatic lipids then their sucrose gavaged counterparts (Figure 3A–C). We next tested whether treatments with refined sugars or natural sweeteners can impact liver inflammation, which is a key driver of NAFLD pathogenesis. We assessed the levels of the cytokines IL-1β, IL-6, and TNF-α as well as the chemokine RANTES since previous studies have shown that they are elevated in liver of diet-induced rodent models of obesity and contribute to NAFLD pathogenesis [33,34]. We found that only IL-1β was increased by HFHS feeding and that most of the natural sweeteners—either maple, molasses, agave, and corn, but also fructose—reduced the hepatic levels of this key pro-inflammatory cytokine compared to sucrose treatment (Figure 3D). No differences between refined and natural sweeteners were observed for hepatic levels of IL-6, TNF-α, and RANTES (Figure 3E–G).

## 4. Discussion

While the vast majority of added sugars are consumed as sucrose, composed of equal parts of glucose and fructose, it is mainly the latter that is believed to represent the main culprit for the obesity and T2D epidemic. Furthermore, less is known about the metabolic impact of chronic consumption of natural sweeteners, which are usually considered only for their sugar content and not for the other nutrients and phytochemicals they also contain. In the present study, we evaluated the metabolic impact of the addition of only 4% of the total daily calories consumed as added sugar coming from natural sweeteners (e.g., from maple, brown rice, agave, and corn syrups, molasses, or honey) as compared to an equivalent amount of sucrose. We found that treatment of HFHS-fed obese rats with most of the types of natural sweeteners reduced insulin resistance compared to sucrose-treated HFHS-fed animals. This extends our previous work, where we showed that a single gavage with maple and agave syrups as well as molasses resulted in a lower blood glucose and insulin responses [28]. We thus propose that daily substitution of refined sucrose with natural sweeteners would generate lower glycemic and insulinemic responses, translating into better metabolic health in the long-term.

Despite the fact that natural sweeteners are often described as just sugar, they actually contain many other nutrients (e.g., vitamins and minerals) as well as many phytochemicals including different classes of polyphenols. The latter molecules have been shown to influence glucose metabolism by several mechanisms. They can inhibit carbohydrate digestion and absorption through inhibiting α-glucosidase [35,36], sodium-dependent glucose transporter 1 [37,38], and sodium-independent glucose transporter [38,39] in the intestine, therefore reducing glucose absorption. Among the natural sweeteners we tested, maple syrup has already been shown to modulate glucose absorption in part due to its polyphenols content. An extract of maple syrup rich in polyphenols (MSX) has been reported to inhibit the activity of α-glucosidase in vitro and to decrease the uptake of glucose in HepG2 cells [40]. Moreover, another team found that administration of maple syrup compared to sucrose in rats reduced plasma glucose levels [22] and intestinal glucose absorption [23]. The reduction of glucose absorption they observed was increased when maple syrup was darker, which was associated with an increased antioxidant activity [23]. Thus, we can hypothesize that chronic inhibition of glucose absorption might have provided an advantage for these animals treated with natural sweeteners compared to those treated with sucrose. It has also been reported that the metabolic benefits of honey cannot be reproduced by a solution that only contains its equivalent sugar content [41,42], which supports the concept that bioactive molecules found in honey and probably other natural sweeteners can counteract the detrimental metabolic effects of added refined sugars. Corn syrup was the only natural sweetener that was not able to significantly reduce insulin resistance compared to sucrose-treated HFHS-fed rats. This lack of effect might be explained by the lower concentration of total polyphenols in corn syrup compared to all the other natural sweeteners tested, as previously reported [28].

Polyphenols have also been proposed to protect from NAFLD (reviewed in [43,44]). We have previously shown that a polyphenol-rich cranberry extract prevented obesity and hepatic steatosis [45] and could even reverse liver steatosis independently from its anti-obesity effect [46]. In the current study, we showed that chronic intake of maple syrup, molasses, agave, and corn syrups was associated with a blunted hepatic inflammation compared to sucrose intake as revealed by a significant and specific reduction of IL-1βprotein level, an important mediator of NAFLD in animal models [47,48] Indeed, mice deficient in IL-1β signaling, or in inflammasome components converting the pro-form of IL-1β to its active form, are resistant to the development of high fat diet-induced hepatic steatosis. Importantly, a recent study comparing the metabolic impact of fructose to sucrose also reported that the disaccharide was inducing higher level of IL-1β in kidney, and was more detrimental on glucose metabolism [49]. It is interesting to put these results using pure maple syrup in line with a previous study evaluating the hepatic effect of 8 weeks supplementation of HF diet with the maple syrup extract, known as MSX, on rats. MSX did not reduce TG or cholesterol accumulation in the liver but mitigated hepatic inflammation caused by the HF diet as shown here suggesting that the anti-inflammatory molecule(s) in maple syrup may be enriched in this extract [50]. It was showed that 17 genes involved in immune response were up-regulated in the liver, six being related to protection against infection. In line with these findings, MSX has also been reported to help antibiotics to fight against resistant bacteria in isolated cells [51].

The present study was also designed to test whether the daily and chronic addition of fructose could indeed exert more deleterious metabolic effects compared to isocaloric amounts of sucrose in a diet induced obesity model. Indeed, in contrast to glucose, it is known that dietary fructose is largely metabolized in the liver where the major part is converted into glucose and then either stored as glycogen or released into the circulation. Consequently, fructose consumption does not immediately stimulate the secretion of insulin [52]. Moreover, part of the fructose pool in the liver will be converted into lactate and a small remaining proportion will be converted into fatty acids. Although minor, this last pathway could nevertheless be implicated in the development of liver steatosis, particularly in a fructose overconsumption context [53]. Indeed, studies have shown that high consumption of fructose, representing 25% of the total energy intake, is associated with NAFLD and dyslipidemia [54,55]. However, when fructose is consumed at lower doses of 18% or 9% of total calories, it was not associated with a greater risk of MetS and CVD [56]. Consistent with this, it has recently been demonstrated that fructose at non-excessive doses is mainly converted to glucose in the enterocyte before its absorption [57]. Fructose is absorbed intact only when it is ingested in an amount that exceeds the capacity of its conversion to glucose in the enterocyte. In the present study, we found that daily chronic fructose administration did not result in greater hepatic steatosis than an equivalent amount of sucrose, as revealed by similar accumulation of lipids in the liver of HFHS-fed obese rats. In contrast to previous studies in rodent models, the added sugars provided by gavage in our study only represented 4% of the total daily energy intake of the rats, and the total fructose consumption, including from the diet, represented ~13% of the total energy intake, which is more relevant to typical human consumption of these added sugars in humans. We found that in these more physiologically and clinically relevant conditions, that fructose is in fact less harmful than an equivalent dose of sucrose. Our findings thus demonstrate that fructose is not metabolized preferentially into fatty acid in the liver compared to glucose when both sugars are given to rats at levels that are more nutritionally relevant to typical human consumption. Nevertheless, it cannot be excluded that the effects we observed are specific to murine metabolism which might differ from the human metabolism. Other limitations of the study should be noted. The duration of the study was 8 weeks and a longer period of treatment with the HFHS diets and the various sweeteners might have resulted in more significant differences. Moreover, the data could result from differences in absorption, as it is known that the presence of both glucose and fructose leads to greater fructose absorption [58]. Finally, further investigations are needed to identify the mechanism of actions underlying the healthy benefits of the natural sugars. Those benefits were furthermore found compared to an equivalent amount of sucrose and future studies are needed to determine if those natural sweeteners also have health benefits versus other simple sugars or dietary conditions.

The population’s awareness of the recommendation of health agencies to limit intake of total added sugar motivates consumers to find alternative solutions. This is in part explaining the success of the low or no calorie non-nutritive sweeteners (NNS), offering a palatable alternative to caloric sugar in all kinds of products [59]. Nevertheless, despite this substitution of NNS at the expense of regular added sugar, the prevalence of obesity and associated diseases continue to rise [60]. Another less harmful alternative might be to improve our understanding of the metabolic impact of natural sweeteners. Indeed, despite the fact that natural sweeteners are rich in sugar and must be consumed with moderation, the present data clearly indicate that these sweeteners should be considered as more than just sugar sources. Importantly, natural sweeteners resulted in improved metabolic health when substituting sucrose even if this represented only 4% of the total daily energy consumed by the animals. Substituting the total intake of refined added sugars by natural sweeteners would not be possible in human as 90% of added sugars intake come from industrial products [7]. However, 4% of total energy intake in humans is equivalent to only 5 teaspoons of sucrose, which substitution with natural sweeteners is clearly achievable.

## Figures and Tables

**Table 1:** Effects of refined sugars and natural sweeteners on body and organs weights.

		Body (g)	Liver (g)	Pancreas (g)	iWAT (g)	eWAT (g)	BAT (g)
Chow	Sucrose	557.3 ± 20.2	14.41 ± 1.02	1.58 ± 0.09	7.95 ± 1.58	11.74 ± 0.86	0.56 ± 0.05
HFHS	Sucrose	650.3 ± 17.9 #	16.23 ± 0.81	1.27 ± 0.06 #	16.45 ± 1.57 #	23.84 ± 1.53 #	0.82 ± 0.03 #
	Fructose	652.2 ± 20.9	16.40 ± 0.81	1.29 ± 0.07	12.00 ± 1.17	21.68 ± 1.53	0.77 ± 0.07
	Maple	633.9 ± 14.8	15.18 ± 0.35	1.36 ± 0.06	14.56 ± 1.47	22.03 ± 1.48	0.63 ± 0.03 **
	Molasses	606.4 ± 13.8	14.82 ± 0.52	1.45 ± 0.04	11.56 ± 1.18	18.23 ± 1.25	0.66 ± 0.04 *
	Brown Rice	633.3 ± 15.9	15.49 ± 0.73	1.36 ± 0.07	15.02 ± 1.55	22.91 ± 1.55	0.74 ± 0.02
	Agave	638.5 ± 17.1	15.32 ± 0.57	1.33 ± 0.06	12.97 ± 1.55	23.65 ± 2.33	0.73 ± 0.06
	Corn	639.3 ± 12.8	15.14 ± 0.72	1.34 ± 0.06	13.96 ± 1.46	20.78 ± 1.08	0.69 ± 0.05
	Honey	621.7 ± 12.6	15.26 ± 0.41	1.49 ± 0.06	12.01 ± 1.56	18.91 ± 1.77	0.63 ± 0.05 *

Body and organs weights after 8 weeks of treatment in male Wistar rats. Data represent mean ± SEM, *n* = 10–12 rats. # *p* < 0.05, *t*-test for chow-sucrose (reference for high fat high sucrose (HFHS)-induced obesity) versus HFHS-sucrose. * *p* < 0.05, ** *p* < 0.01, one-way ANOVA for fructose and natural sweeteners versus HFHS-sucrose. BAT, brown adipose tissue; eWAT, epididymal white adipose tissue; iWAT, inguinal white adipose tissue.

**Table 2:** Effects of refined sugars and natural sweeteners on respiratory exchange ratio.

		O_2_ 24 h (mL/kg^0.75^/h)	CO_2_ 24 h (mL/kg^0.75^/h)	EE 24 h (cal/h/g^0.75^)	RER 24 h
Chow	Sucrose	811 ± 8	772 ± 17	22.8 ± 0.4	0.95 ± 0.01
HFHS	Sucrose	770 ± 13 #	638 ± 13 ###	21.2 ± 0.4 #	0.82 ± 0.00 ###
	Fructose	811 ± 14	670 ± 14	22.1 ± 0.4	0.83 ± 0.00
	Maple	793 ± 8	647 ± 7	21.5 ± 0.2	0.82 ± 0.00
	Molasses	813 ± 19	653 ± 13	22.1 ± 0.5	0.80 ± 0.00
	Brown Rice	756 ± 14	616 ± 16	20.6 ± 0.4	0.78 ± 0.03
	Agave	756 ± 44	654 ± 10	21.6 ± 0.3	0.82 ± 0.01
	Corn	780 ± 12	650 ± 17	21.2 ± 0.3	0.83 ± 0.01
	Honey	798 ± 39	669 ± 21	22.3 ± 0.7	0.82 ± 0.01

Average energy expenditure and respiratory exchange ratio over 24 h after 6 weeks of treatment in male Wistar rats. Data represent mean ± SEM, *n* = 8–10 rats. # *p* < 0.05, ### *p* < 0.001, *t*-test for chow-sucrose (reference for HFHS-induced obesity) versus HFHS-sucrose. One-way ANOVA for fructose and natural sweeteners versus HFHS-sucrose (no significant change was detected). CO2, volume of carbon dioxide produced; EE, energy expenditure; O2, volume of oxygen consumed; RER, respiratory exchange ratio.
